# Machine Learning Models for Sepsis: From Early Detection to Short- and Long-Term Prognosis

**DOI:** 10.3390/ijms27062721

**Published:** 2026-03-17

**Authors:** Maria Vittoria Ristori, Filippo Ruffini, Silvia Spoto, Roberto Cammarata, Vincenzo La Vaccara, Lucrezia Bani, Damiano Caputo, Paolo Soda, Valerio Guarrasi, Silvia Angeletti

**Affiliations:** 1 National PhD Program in One Health Approaches to Infectious Diseases and Life Science Research, Department of Public Health, Experimental and Forensic Medicine, University of Pavia, 27100 Pavia, Italy; mariavittoria.ristori01@universitadipavia.it; 2 Research Unit of Clinical Laboratory Science, Department of Medicine and Surgery, Università Campus Bio-Medico di Roma, Via Alvaro del Portillo, 21, 00128 Roma, Italy; 3 Operative Research Unit of Laboratory, Fondazione Policlinico Universitario Campus Bio-Medico, Via Alvaro del Portillo, 200, 00128 Rome, Italy; 4 Research Unit of Artificial Intelligence and Computer Systems, Department of Engineering, Università Campus Bio-Medico di Roma, Via Alvaro del Portillo, 21, 00128 Roma, Italy; filippo.ruffini@unicampus.it (F.R.); p.soda@unicampus.it (P.S.); 5 Diagnostic and Therapeutic Medicine Department, Fondazione Policlinico Universitario Campus Bio-Medico, Via Alvaro del Portillo, 200, 00128 Rome, Italy; s.spoto@policlinicocampus.it; 6 Operative Research Unit of General Surgery, Fondazione Policlinico Universitario Campus Bio-Medico, 00128 Rome, Italy; roberto.cammarata@policlinicocampus.it (R.C.); v.lavaccara@policlinicocampus.it (V.L.V.); lucreziabani3@gmail.com (L.B.); d.caputo@policlinicocampus.it (D.C.); 7 Department of Diagnostics and Intervention, Radiation Physics, Biomedical Engineering, Umeå University, 901 87 Umeå, Sweden

**Keywords:** Artificial Intelligence, biomarkers, risk stratification, prognosis, explainable AI, clinical decision support systems

## Abstract

Sepsis is a leading cause of morbidity and mortality worldwide, and its outcomes depend on early recognition and timely intervention. Conventional clinical scores and biomarkers provide prognostic value but often lack accuracy for individualized prediction. Machine learning (ML) offers the ability to integrate multidimensional data to improve risk stratification. We analyzed 477 patients admitted to our hospital, including 251 with sepsis, 100 with septic shock, and 126 controls. Demographic, clinical, and laboratory data were collected. Univariate correlation analyses explored associations with sepsis severity and mortality (in-hospital, 30-day, and 90-day). Several ML models were tested, with performance assessed by area under the receiver operating characteristic curve (AUC-ROC) and Matthews’s correlation coefficient (MCC). Model interpretability was evaluated using SHAP (SHapley Additive exPlanations). Sepsis severity and mortality correlated with biomarkers (procalcitonin, mid-regional pro-adrenomedullin, lactate) and clinical scores (SOFA, qSOFA). In-hospital mortality was associated with ADM, catecholamine use, and SOFA, while 90-day mortality involved smoking and Gram-negative or polymicrobial infections. Different machine learning models were evaluated, and the model achieving the highest performance on the validation set was selected. The selected model either outperformed or demonstrated comparable performance to logistic regression, depending on the specific prediction task (AUC 0.99 for sepsis, 0.96 for septic shock, 0.70 for ICU admission; 0.90, 0.72, and 0.87 for in-hospital, 30-day, and 90-day mortality). SHAP confirmed the clinical relevance of these predictors. ML models integrating clinical and biochemical data outperform conventional methods in predicting sepsis progression and mortality, while maintaining interpretability. These findings support the use of ML-based tools for early diagnosis and personalized risk stratification in sepsis, though external validation is required before clinical application.

## 1. Introduction

Sepsis represents one of the most critical challenges in modern medicine, arising from a maladaptive host response to infection that can rapidly progress to multi-organ dysfunction or death [1]. With contempt improvements in intensive care and therapeutic strategies, sepsis remains a leading cause of mortality worldwide, largely due to difficulties in its timely and accurate diagnosis [2]. The heterogeneous nature of its clinical presentation, together with overlapping features shared with other inflammatory conditions, often hinders early recognition and delays treatment initiation [3]. Conventional diagnostic approaches, including physical examination and routine laboratory analyses, are frequently insufficient because of their limited sensitivity and specificity, underscoring the need for more robust diagnostic tools [4,5]. Over the past decade, circulating biomarkers has gained increasing attention as adjuncts to clinical evaluation. Molecules such as C-reactive protein (CRP), procalcitonin (PCT), Mid-Regional pro-ADrenoMedullin (MR-proADM), interleukin-6 (IL-6), and lactate have been widely studied for their ability to reflect systemic inflammation, tissue injury, and organ dysfunction [6]. While these biomarkers offer valuable insights into the biological processes underlying sepsis, their stand-alone diagnostic capacity is restricted. For instance, CRP is elevated in a broad range of inflammatory disorders [6], and PCT may remain within normal limits in the early stages of infection [7]. Consequently, single biomarker measurements often provide incomplete information, requiring careful interpretation within the broader clinical context.

To overcome these limitations, Artificial Intelligence (AI) and Machine Learning (ML) has emerged as a promising approach to enhance biomarker-based diagnostics [8,9]. By integrating biomarker levels with other patient-specific parameters such as demographics, comorbidities, vital signs, and laboratory data, ML algorithms can detect complex, non-linear relationships that traditional methods cannot easily capture [10]. ML models can learn from large clinical datasets, identifying subtle patterns that may signal the onset of sepsis before overt clinical symptoms develop [11]. Importantly, ML-based tools can adapt predictions to individual patients, enabling a more personalized diagnostic process, and reducing the risk of misclassification. Recent studies have demonstrated that ML algorithms can improve both the sensitivity and specificity of sepsis detection, in some cases predicting its onset hours before clinical recognition [9,12]. However, challenges remain regarding data quality, bias in training datasets, and the interpretability of advanced models’ issues that must be addressed before widespread clinical adoption [13]. This study aims to examine the evolving role of serum biomarkers in sepsis diagnosis, with particular emphasis on how ML-driven approaches can enhance their diagnostic value. We discuss the strengths and weaknesses of current biomarkers, outline the principles of ML in this context, and explore how integrating these strategies may contribute to earlier and more accurate identification of sepsis, ultimately improving patient management and outcomes.

## 2. Results

This study is a retrospective analysis based on the data from patients with a suspected sepsis, to develop a machine learning model to accurately predict sepsis, septic shock, recovery at Intensive Care Unit (ICU), in-hospital mortality as well as mortality at 30 and 90 days in patients.

We designed the analysis to follow the natural progression of sepsis severity and patient mortality outcomes. We first evaluated variables directly related to the acute presentation and management of the disease, such as presence of sepsis, presence of septic shock, use of catecholamines, and admission to the intensive care unit, that reflect the immediate clinical, therapeutic intensity, and early prognostic indicators in the septic population.

These are crucial for understanding the initial severity profile and treatment requirements, which are strongly associated with short-term outcomes. Subsequently, we extended our analysis to in-hospital mortality, 30-day mortality, and 90-day mortality, to explore how these early clinical features and management strategies translated into patient survival over different time points. This stepwise approach allowed us to identify not only acute predictors of severity but also longer-term prognostic factors, providing a more comprehensive understanding of outcome trajectories in sepsis patients.

The global dataset includes 477 patients, among which 251 septic patients, 100 septic shocks and 126 control cases. For each patient, we collected multiple variables, including demographic characteristics, clinical data, clinical scores and laboratory biomarkers, as described in the previous paragraph (Table 1). Our study included 251 patients with sepsis, 100 patients with septic shock, and 126 controls. Of these, 47 patients were admitted to the intensive care unit, and 89 patients required catecholamine support (CCS). There were 160 cases in the death group, of which 43 died during the hospitalization, 48 30-day Mortality and 69 90-day Mortality; 191 cases were in the survival group.

### 2.1. Univariate Correlation Analysis

**Figure 1:**
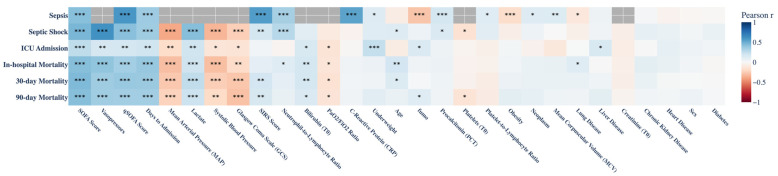
Correlation matrix showing association between clinical, biochemical, and molecular parameters in three clinically relevant subgroups presented as different rows: sepsis, septic shock, ICU Admission, in-hospital mortality, 30-day mortality, 90-day mortality. Red and blue colors denote negative and positive correlations, respectively, whereas gray cells indicate features that were unavailable in the original dataset and therefore excluded from the corresponding analysis. The intensity of color and asterisks denote statistical significance (* *p* < 0.05, ** *p* < 0.01, *** *p* < 0.001).

We performed an explorative analysis of our dataset by using a univariate analysis with six correlation matrices (Presence of Sepsis, Presence of Septic Shock, Admission to Intensive Care Unit, Intra-hospital Mortality, 30-day Mortality and 90-day Mortality). Each matrix shows the correlation between clinical variables and a specific outcome. First, we considered the correlation with sepsis-related conditions (Figure 1). Several variables demonstrated significant correlations with the presence of sepsis. Strong positive correlation was observed between the presence of sepsis and biomarkers, such as ADM, PCT (procalcitonin), CRP (C-reactive protein), NLR (neutrophil-to-lymphocyte ratio), clinical score, such as SIRS score, SOFA score, qSOFA score and smoking status. PLR (platelet-to-lymphocyte ratio), MCV (Mean Corpuscular Volume), neoplasia and underweight highlighted a low correlation. Pneumopathy, obesity and smoke showed an inverse correlation. Other comorbidities such as cardiopathy, liver disease, chronic kidney disease, and diabetes did not show significant correlations with sepsis in this analysis.

The second row of Figure 1 presents a complex correlation pattern in patients with septic shock. Lactate, ADM, and NLR levels correlated strongly with clinical severity indicators. Also, clinical scores (SOFA, qSOFA), use of catecholamines, and Gram negative infectious showed strong positive correlation. Age, PCT, SIRS, and smoke highlighted a poor correlation. Furthermore GCS, MAP, and PAS showed a strong negative correlation. Other variables did not have a correlation with septic shock. The third row in Figure 1 shows the correlations results in the subgroup of patients requiring ICU admission. Only underweight and SOFA score showed a strong positive correlation. Biomarkers of systemic inflammation and organ failure, such as lactate, ADM, and bilirubin, showed a positive correlation with ICU admission status. Notably, epathopatia and use of catecholamine maintained their association across all subgroups, underscoring their consistency as severity markers. Parameters such as albumin and lymphocyte count were inversely correlated with ICU admission, in line with the known catabolic and immunosuppressive effects of critical illness. In the fourth row of the matrix in Figure 1, in-hospital mortality significant strong positive correlations (*p* < 0.001) were observed in laboratory parameters, such as ADM and catecholamines. Also, clinical scores (SOFA and qSOFA) showed a significant strong positive correlation (*p* < 0.001). Other variables, such as age, PCT, bilirubin, pneumopathies, and microbiology data (Gram negative infection) highlighted a positive correlation (*p* < 0.05 and *p* < 0.01). Conversely, negative correlations were identified with parameters such as GCS, PO2/FIO2 ratio, MAP and PAS. For 30-day mortality (fifth row in Figure 1), a similar pattern emerged as for in-hospital mortality, with the addition of the Sirs descriptor. For 90-day mortality (last row in Figure 1), the correlation profile largely mirrored that of in-hospital and 30-day mortality, with significant positive associations for severity scores and inflammatory markers, with the addition of smoke and microbial-related variables (gram negative and polymicrobial infection).

### 2.2. ROC Curve Analysis and SHAP Interpretation for Septic and Mortality Outcomes

**Figure 2:**
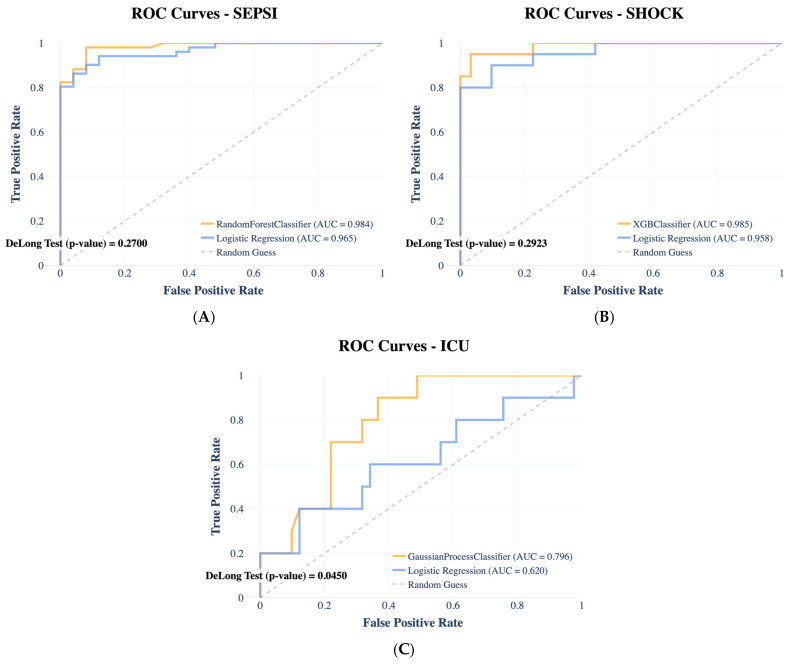
ROC curves comparing the performance of logistic regression (LR) and the selected ML model over three prediction tasks: (**A**) Random Forest for Sepsi prediction, (**B**) XGBoost Classifier for Shock prediction, and (**C**) Gaussian Process Classifier for ICU admission prediction.

**Figure 3:**
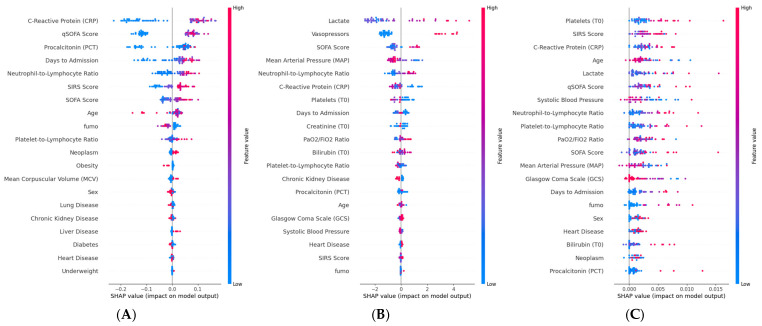
SHAP summary plots, showing the impact of variables on three different prediction outputs obtained by the best ML models: (**A**) The most relevant features to predict the sepsis by the Random Forest Classifier algorithm. (**B**) The most relevant features to predict septic shock by the XGBoost Classifier algorithm using the dataset. (**C**) The most relevant features to predict recovery at Intensive Care Unit (ICU) by the Gaussian Process Classifier using the dataset.

For each prediction task, the best-performing model was independently selected based on MCC evaluated on the validation set. The resulting selected models are Random Forest Classifier for sepsis prediction, XGBoost Classifier for septic shock prediction, and Gaussian Process Classifier for ICU admission prediction. We report model accuracy in terms of area under the ROC curves (Figure 2), computed on the held-out test set. The AUC values for (A) sepsis prediction, (B) shock prediction, and (C) ICU admission prediction are 0.99, 0.96, and 0.70, respectively. Across all prediction tasks, the selected ML models outperformed logistic regression, with AUC values of 99%, 98.5%, and 79% for sepsis, shock, and ICU admission. ROC curves were plotted to illustrate the diagnostic performance across different threshold levels. Additionally, SHAP was employed to analyze the contribution of individual variables to model predictions. The results, including summary plots, are shown in Figure 3.

For sepsis prediction (Figure 3A), the Random Forest Classifier identified CRP as the most influential feature, with higher values strongly pushing predictions toward the sepsis class. qSOFA score ranked second, followed by PCT and days to admission. The neutrophil-to-lymphocyte ratio (NLR) and SIRS score also contributed substantially to predictions, while SOFA score appeared among the mid-ranked features. Age and smoking status showed modest but visible effects. Comorbidities such as lung disease, chronic kidney disease, and diabetes had minimal influence on sepsis classification. For septic shock prediction (Figure 3B), the XGBoost Classifier assigned the highest importance to lactate, with elevated values strongly associated with positive predictions. Vasopressor use and SOFA score were the second and third most influential features, followed by mean arterial pressure, where lower values drove predictions toward shock. NLR and CRP contributed to the model at an intermediate level, while creatinine, PaO_2_/FiO_2_ ratio, and bilirubin reflected the role of organ dysfunction in shock identification. GCS and systolic blood pressure showed inverse associations, consistent with their known relationship to hemodynamic compromise. For ICU admission prediction (Figure 3C), the Gaussian Process Classifier yielded a distinct feature importance profile. Platelet count emerged as the most influential variable, followed by SIRS score and CRP. Age and lactate contributed at an intermediate level, while qSOFA score and systolic blood pressure also showed visible effects. The overall SHAP magnitudes were considerably smaller compared to sepsis and shock models, consistent with the lower discriminative performance observed for this endpoint (AUC = 0.70). Underweight status, while previously noted in the correlation analysis, ranked among the lower-impact features in the SHAP analysis, suggesting that its predictive contribution is partially captured by other covariates in the multivariate model.

**Figure 4:**
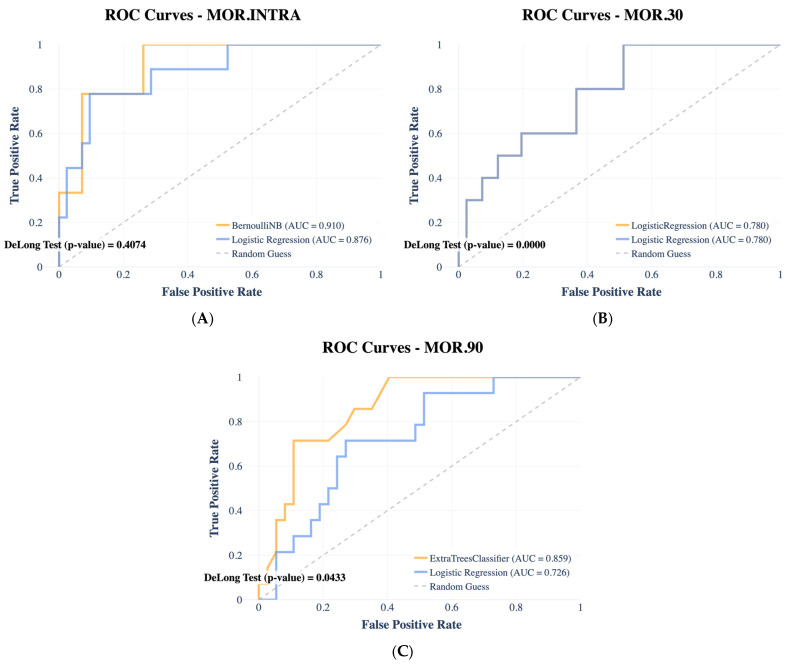
ROC curves comparing the performance of Logistic Regression (LR) model and the selected best ML model (orange) over three prediction tasks: (**A**) Bernoulli Naïve Bayes classifier for In-hospital mortality, (**B**) Logistic Regression for 30-day mortality prediction, and (**C**) Extra Tree Classifier for 90-day mortality prediction.

**Figure 5:**
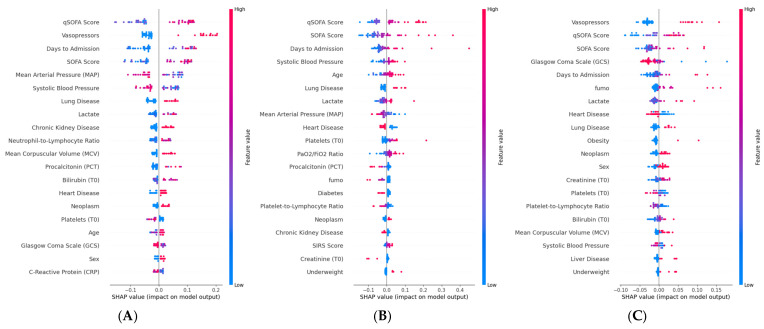
SHAP summary plots, showing the impact of variables on three different prediction outputs obtained by the best ML models: (**A**) The most relevant features to predict the in-hospital mortality by BernoulliNB model using the dataset. (**B**) The most relevant features to predict 30-day mortality by the Logistic Regression model using the dataset. (**C**) The most relevant features to predict 90-day mortality by the ExtraTreeClassifier model using the dataset.

Following the same model selection procedure described for the septic outcomes, the best-performing model was independently selected for each mortality endpoint based on MCC evaluated on the validation set. The resulting selected models are Bernoulli Naïve Bayes for in-hospital mortality, Logistic Regression for 30-day mortality, and Extra Trees Classifier for 90-day mortality. We report model accuracy in terms of area under the ROC curves (Figure 4), computed on the held-out test set. The AUC values for (A) in-hospital mortality, (B) 30-day mortality, and (C) 90-day mortality are 0.91, 0.78, and 0.86 respectively. Notably, for 30-day mortality, Logistic Regression achieved the highest MCC among all 25 candidate algorithms, indicating that a linear decision boundary was sufficient to capture the prognostic signal for this endpoint. ROC curves were plotted to illustrate diagnostic performance across different threshold levels, and the DeLong test was used to assess the statistical significance of differences between each selected model and the baseline Logistic Regression. We also conducted SHAP analysis for mortality outcomes (Figure 5). For in-hospital mortality (Figure 5A), the BernoulliNB model identified qSOFA score and vasopressor use as the most influential predictors, followed by days to admission, SOFA score, and hemodynamic variables (MAP, systolic blood pressure), with lower values driving predictions toward higher risk. Lactate showed a positive association with mortality, while preserved GCS was protective. For 30-day mortality (Figure 5B), the Logistic Regression model assigned the highest importance to qSOFA and SOFA scores, followed by days to admission and systolic blood pressure. Age gained prominence compared to the in-hospital setting, consistent with the increasing influence of baseline patient characteristics on post-discharge outcomes. For 90-day mortality (Figure 5C), the ExtraTreeClassifier highlighted vasopressor use and qSOFA as dominant predictors, followed by SOFA and GCS. Smoking status and days to admission gained prominence relative to shorter-term endpoints, reflecting the growing role of lifestyle factors in long-term prognosis. Comorbidities including heart disease, lung disease, and neoplasm also contributed, suggesting that comorbidity burden becomes increasingly relevant for late mortality.

### 2.3. Effect of Clinical Severity Scores on Predictive Performance

To evaluate the potential impact of feature–label circularity, we performed a sensitivity analysis in which severity scores were systematically removed from the input space. The same model families and identical train/test splits were maintained; only the feature set was modified. Specifically, SOFA, qSOFA, and SIRS were excluded for sepsis, while for shock we additionally removed variables directly embedded in the diagnostic criteria.

**Figure 6:**
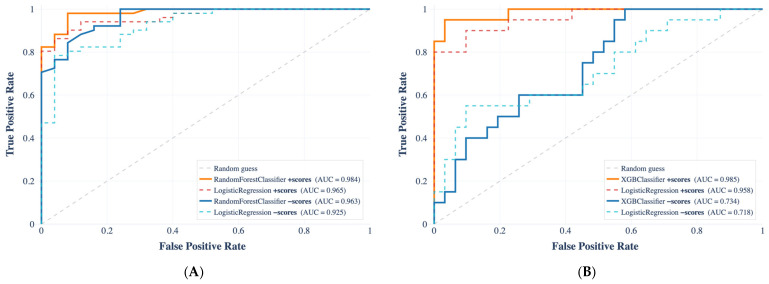
ROC curves for (**A**) Sepsis and (**B**) Shock classification under an input-variable ablation of clinical severity scores SOFA, qSOFA, and SIRS. Orange curves report performance when these scores are included, whereas blue curves correspond to the ablated setting where the score variables are removed from the input. For each task, we display the best-performing model selected in this Random Forest Classifier and XGBoost for Sepsi and Shock, respectively.

Figure 6 reports the corresponding ROC curves for the best-performing model selected in the primary analysis (Random Forest for sepsis and XGBoost (version 3.2.0) for shock). For sepsis (Figure 6A), excluding SOFA/qSOFA/SIRS produced a limited reduction in discrimination. The AUC decreased from 0.984 to 0.963, indicating that most of the predictive signal is retained even after removal of the severity scores. Although discrimination remains high in the ablated setting, the visible separation between curves confirms that inclusion of score-related variables provides an incremental performance gain. In contrast, for shock (Figure 6B), ablation resulted in a marked deterioration in performance. The AUC declined from 0.985 to 0.734, with a clear divergence between the full-feature and ablated ROC curves across the entire false-positive rate range. This substantial drop indicates that a large fraction of the discriminative capacity in the full model is attributable to variables embedded in the shock definition. Overall, the ROC analysis highlights a moderate dependence on severity scores for sepsis prediction and a pronounced dependence for shock prediction, underscoring the importance of accounting for definition-related features when interpreting model performance.

### 2.4. Pilot Clinical Validation and Decision Curve Analysis

**Figure 7:**
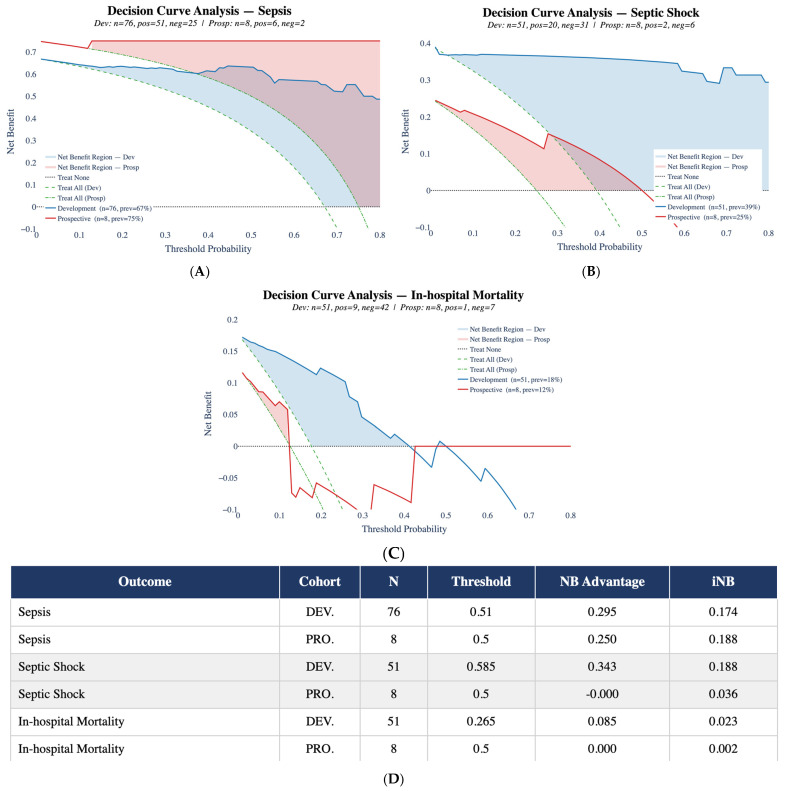
Decision Curve Analysis for (**A**) sepsis, (**B**) septic shock, and (**C**) in-hospital mortality in the development (DEV) and prospective (PRO) cohorts. Net benefit is plotted across threshold probabilities and compared with default management strategies (“treat all” and “treat none”). The table (**D**) summarizes optimal thresholds, net benefit (NB) and incremental Net Benefit (iNB).

Decision curve analyses were conducted for sepsis, septic shock, and in-hospital mortality in both the development (DEV) and prospective cohorts (PRO), as displayed in Figure 7. For sepsis prediction in Panel 7A, the development cohort showed a maximum net benefit of 0.295 at a threshold probability of 0.51, corresponding to an incremental net benefit of 0.174. In the prospective cohort, the optimal threshold was 0.50, with a net benefit of 0.250 and an incremental net benefit of 0.188. Across the evaluated range of threshold probabilities, the model’s net benefit remained above both the “treat all” and “treat none” strategies in both cohorts. For septic shock prediction in Panel 7B, the development cohort demonstrated a maximum net benefit of 0.343 at a threshold of 0.585, with an incremental net benefit of 0.188. In the prospective cohort, the optimal threshold was 0.50, with a net benefit approximately equal to zero and an incremental net benefit of 0.036. The net benefit curve in the prospective cohort overlapped with default strategies across most threshold values. For in-hospital mortality prediction in Panel 7C, the development cohort achieved a maximum net benefit of 0.085 at a threshold of 0.265, corresponding to an incremental net benefit of 0.023. In the prospective cohort, the optimal threshold was 0.50, with net benefit approximately equal to zero and incremental net benefit of 0.002. In this setting, the net benefit curve showed minimal separation from default strategies.

A summary of optimal threshold probabilities, net benefit values, incremental net benefit, and resulting clinical recommendation status for each outcome and cohort is presented in Panel 7D. Notably, sepsis prediction was the only endpoint for which the model yielded a positive net benefit advantage in both the development and prospective cohorts. For septic shock and in-hospital mortality, the prospective cohort showed near-zero or negative net benefit values, indicating limited clinical added value over default management strategies in this small external sample.

## 3. Discussion

A large multicenter analysis in 27 academic hospitals reported a marked rise in the incidence of septic shock [14,15], increasing from 12.8 to 18.6 cases per 1000 hospital admissions. During the same period, mortality rates showed only a modest decline, from 55% to 51% [16]. This growing burden has been linked to multiple contributing factors, including the aging of the population, higher rates of immunosuppression, and the spread of multidrug-resistant pathogens, emphasizing the persistent threat of sepsis as a major global health problem [17,18]. Although conventional inflammatory biomarkers remain central to the clinical diagnosis of sepsis, there is still a substantial lack of research focusing on immune exhaustion in these patients [19], a gap that may contribute to both insufficient and excessive treatment strategies [20,21]. More studies are trying to delve deeper and find biomarkers for the prognosis of sepsis and septic shock to improve the quality of care and positive outcomes of hospitalized patients [6]. In this framework, the use of machine learning models (ML) to predict sepsis or septic shock and their outcomes are increasing, to enter in clinical practice [22]. In response to these challenges, we developed a multi-biomarker model using ML techniques to predict sepsis, septic shock, ICU admission, and mortality outcomes in a real-world clinical cohort. By combining demographic, clinical, and biomarker data, we demonstrated that ML algorithms substantially outperform traditional logistic regression models in discriminating septic conditions and predicting outcomes across multiple time points.

Previous studies have reported improved diagnostic performance of machine learning models compared with traditional statistical approaches in sepsis prediction, with AUC values typically ranging between 0.75 and 0.95 depending on the dataset, timing of prediction, and variables included [8,11,23,24]. In line with these reports, our models achieved AUC values of 0.99 for sepsis and 0.96 for septic shock, confirming the strong discriminative potential of ML approaches in high-dimensional clinical data. However, beyond diagnostic classification, our study expands current evidence by simultaneously addressing short- and long-term prognostic endpoints (ICU admission, in-hospital, 30-day, and 90-day mortality) within a single integrated modeling framework.

To further contextualize these performance estimates, we performed a sensitivity analysis evaluating the potential impact of feature–label circularity on model discrimination. Clinical severity scores such as SOFA, qSOFA, and SIRS are integral components of the Sepsis-3 diagnostic criteria [1] and were simultaneously used as input variables in our predictive models. This overlap raises the possibility that observed discriminative performance may partly reflect rule-based re-identification of diagnostic labels rather than genuinely independent prediction [8]. To address this concern, severity scores were systematically removed from the input feature space while maintaining identical model families and train/test splits.

For sepsis prediction (Figure 4A), exclusion of SOFA, qSOFA, and SIRS produced only a limited reduction in discrimination, with the AUC decreasing from 0.984 to 0.963. This finding indicates that a substantial portion of the predictive signal is retained by variables independent of the formal diagnostic criteria, including biomarkers such as PCT, MR-proADM, and CRP, as well as routinely available demographic and clinical parameters. From a clinical perspective, this result suggests that admission-level features may support early risk stratification even before severity scores are fully computed, a scenario of relevance in emergency department settings where timely decision-making is critical [3,9].

In contrast, shock prediction (Figure 4B) exhibited a pronounced degradation in performance following feature ablation, with the AUC declining from 0.985 to 0.734. The marked separation between the full-feature and ablated ROC curves across the entire false-positive rate range indicates that a large fraction of the discriminative capacity in the complete model is attributable to variables embedded in the shock definition, including hemodynamic parameters and vasopressor use. From a clinical standpoint, this finding implies that models incorporating these features may largely reproduce established diagnostic rules rather than provide genuinely anticipatory prediction of hemodynamic deterioration. This observation is consistent with previous reports highlighting the risk of tautological prediction in sepsis-related machine learning studies, where features derived from clinical workflows or scoring systems can artificially inflate model performance [8,25].

A key strength of our analysis lies in the progressive evaluation of outcome trajectories, to try to reflect the natural course of sepsis. We first focused on acute severity indicators, such as sepsis diagnosis, septic shock, ICU admission, and use of catecholamine use. After we extend the analysis to mortality at different follow-up intervals, in-hospital mortality, 30-day mortality and 90-day mortality. We want to evaluate mortality by 30 days, as it more directly reflects the impact of infection and acute management and reduces the possibility that factors unrelated to the infection (e.g., other comorbidities, intercurrent events) could influence the outcome [26], as might happen in 90-day mortality. In contrast, the assessment of 90-day mortality makes it possible to capture the tail of infection-related events (e.g., sequelae, late complications, secondary infections), providing a more ‘realistic’ picture of the overall burden of the infectious episode, although there is a higher risk of statistical noise since other causes of death not directly related to the infection may come into play [27]. This approach enabled us to delineate both early markers of severity and longer-term prognostic factors. For instance, PCT, MR-proADM, and CRP emerged as crucial drivers of sepsis prediction, while lactate and use of catecholamine were particularly influential in the context of septic shock. Previous studies have extensively documented the association between these biomarkers and sepsis severity and are known to reflect the state of systemic inflammation, hemodynamic instability, and tissue hypoperfusion, all central factors in the pathophysiology of sepsis [7,28,29,30]. Lactate is a well-established marker of tissue hypoperfusion and has consistently been associated with disease severity and mortality in septic shock [31].

Inflammatory indices such as CRP and PCT were significantly associated with the presence of shock, supporting their role in identifying patients at risk of hemodynamic collapse [6]. Also, SOFA and qSOFA scores demonstrated strong correlations, validating their clinical utility in prognostic stratification [32]. SHAP analysis confirmed the clinical interpretability of our ML models. For sepsis prediction, PCT was the most influential variable, followed by MR-proADM, CRP, and established clinical severity scores such as SOFA and qSOFA [33]. In contrast, lactate and catecholamine support were the dominant predictors of septic shock, highlighting the role of metabolic dysfunction and cardiovascular failure in disease progression [34]. Interestingly, underweight status was identified as an influential predictor of ICU admission, which may reflect the vulnerability of malnourished patients to rapid clinical deterioration [35]. These findings underscore the capacity of ML models not only to enhance predictive performance but also to reveal clinically meaningful associations that may inform patient stratification and risk assessment [36]. The correlation analysis of mortality outcomes in septic patients provides valuable insights into the clinical and biological factors that contribute to short- and long-term prognosis [1]. Our findings indicate that both in-hospital and post-discharge mortality are strongly associated with markers of disease severity and systemic inflammation. Specifically, for in-hospital mortality, high levels of ADM and catecholamine support emerged as the most prominent correlates, alongside elevated SOFA and qSOFA scores [37]. These results reinforce the well-established role of hemodynamic instability and organ dysfunction as central determinants of early sepsis-related deaths [38]. The observed associations with additional variables such as PCT, bilirubin, and Gram-negative infections further support the importance of systemic inflammation, impaired liver function, and pathogen-related factors in driving acute mortality risk [6]. Conversely, negative correlations with GCS, PO_2_/FiO_2_ ratio, MAP, and PAS reflect the protective role of preserved neurological, respiratory, and circulatory function in reducing early mortality [39]. The analysis of 30-day mortality revealed a correlation pattern that closely mirrored in-hospital outcomes, but with the additional contribution of the SIRS score. This suggests that systemic inflammatory burden [40], as captured by SIRS criteria, may extend its prognostic relevance beyond the acute phase of hospitalization, continuing to influence short-term outcomes after discharge [41]. By contrast, the 90-day mortality profile emphasized not only the persistence of associations with clinical severity scores and inflammatory biomarkers, but also the growing importance of host and microbial-related factors [42]. Notably, smoking status and the presence of Gram-negative or polymicrobial infections were more strongly linked to late mortality [43]. This finding suggests that long-term prognosis in sepsis survivors is shaped by a combination of baseline vulnerability, lifestyle risk factors, and the complexity of the infectious burden [44]. The progressive involvement of microbial characteristics over time highlights the potential impact of persistent or recurrent infections, multidrug-resistant organisms, and altered host–pathogen interactions on long-term survival [45].

When assessing mortality outcomes, our models achieved strong discriminative ability, with AUC values of 0.90 for in-hospital mortality, 0.72 for 30-day mortality, and 0.87 for 90-day mortality. The lower performance at 30 days may reflect the complex interplay of post-discharge factors, including secondary infections, comorbidities, and treatment adherence, which are not fully captured by baseline clinical and laboratory parameters [46]. SHAP analysis further revealed that MR-proADM and SOFA score were the strongest predictors of in-hospital mortality [47], while qSOFA and catecholamine use gained importance for long-term outcomes [48]. These results emphasize the clinical relevance of these scoring systems in identifying sepsis [1], and highlight the potential of biomarkers for sepsis as valuable clinical tools for assessing disease severity to be used in combination with clinical scores [1,7,28]. Taken together, these results underscore the dynamic nature of sepsis prognosis, where acute mortality is predominantly driven by hemodynamic collapse and multiorgan dysfunction [49], while later outcomes increasingly reflect the interplay between systemic inflammation, infection type, and patient-specific vulnerabilities [42]. These insights have important clinical implications: early recognition and management of hemodynamic failure remain critical for reducing in-hospital mortality, while strategies aimed at optimizing infection control, monitoring long-term inflammatory status, and addressing modifiable risk factors such as smoking may be essential for improving long-term survival [44]. The final predictive modeling task aimed at stratifying patients at risk for sepsis, septic shock, and in-hospital mortality provides important insights into both the strengths and limitations of our machine learning approach. For sepsis prediction, the model demonstrated excellent discriminative capacity, correctly identifying all positive cases. Interestingly, procalcitonin (PCT) emerged as the dominant feature driving predictions, underscoring its clinical relevance as an early biomarker of sepsis and validating previous evidence supporting its diagnostic value [50]. In the case of septic shock prediction, the model successfully classified the most positive patients with high predicted probabilities, highlighting its sensitivity in detecting severe progression [8]. However, the occurrence of two false positives with very high confidence scores suggests a tendency toward overestimation in certain cases. This finding indicates that while the model is robust in identifying true cases of shock, additional refinement and calibration are necessary to improve specificity and minimize the risk of unnecessary escalation of care [11]. Incorporating longitudinal data or combining static biomarkers with dynamic clinical variables may help address this limitation [51]. For in-hospital mortality, the model achieved satisfactory performance, correctly classifying most patients. Notably, the predictions were generally conservative, with low average probabilities assigned to the positive class. This conservative behavior may reduce the likelihood of false alarms but could also limit the model’s ability to detect high-risk patients in real time [9]. Future optimization should aim to balance sensitivity and specificity, ensuring that patients at genuine risk of adverse outcomes are not overlooked [52]. From a clinical perspective, our findings have several implications. First, ML models can be integrated into electronic health records (EHRs) to provide real-time risk assessment, aiding in early diagnosis and individualized treatment planning. Second, the identification of distinct feature sets across different outcomes suggests that tailored predictive tools may be required for specific clinical endpoints. For example, PCT and CRP may be most useful for early detection, while MR-proADM and lactate are more relevant for prognostication. Third, the interpretability provided by SHAP analysis increases clinicians’ trust in ML-based decision support systems, addressing a major barrier to adoption in healthcare. Nevertheless, several limitations must be acknowledged. The retrospective design of our study introduces potential biases related to missing data, unmeasured confounders, and variations in clinical practice. The relatively modest sample size (*n* = 477) may limit the generalizability of our findings, particularly for subgroup analyses.

While the models demonstrated strong discriminative performance in the development cohort, the prospective validation revealed notable differences when clinical utility was assessed through Decision Curve Analysis. Although AUC values remained relatively high for sepsis and septic shock, net benefit analysis provided a more nuanced evaluation of how well the models generalize to new clinical settings. Sepsis prediction maintained a positive net benefit in both development and prospective cohorts, suggesting that its clinical value is preserved across different patient groups, though the limited size of the prospective cohort (*n* = 8). In contrast, septic shock and in-hospital mortality prediction showed a reduction in net benefit in the prospective cohort, despite acceptable discrimination metrics. This observation is in line with a growing body of literature suggesting that statistical accuracy alone does not guarantee clinical usefulness when models are tested on independent populations [53]. Decision Curve Analysis may therefore serve as a useful complementary tool for evaluating real-world applicability, as it accounts for the trade-off between false-positive and false-negative decisions at clinically relevant thresholds. Taken together, these preliminary findings suggest that external validation should go beyond discrimination measures to include assessments of clinical impact, although larger prospective cohorts will be needed to draw firm conclusions regarding readiness for clinical implementation.

### Limitations and Future Studies

Several limitations should be considered. The control group consisted of hospitalized non-septic patients rather than healthy subjects. Although this design may introduce selection bias, it reflects real-world diagnostic conditions in which clinicians must distinguish sepsis from other acute illnesses in hospitalized populations. A potential source of circularity should also be acknowledged, as SOFA and qSOFA scores were used both to define sepsis (Sepsis-3 criteria) and as input variables in the predictive models. While consistent with clinical practice, this may partially influence performance estimates. Future studies should evaluate models excluding severity scores to better assess the independent contribution of biomarkers.

Importantly, the prospective validation revealed differences in clinical transferability when assessed through Decision Curve Analysis. Although discrimination remained high, septic shock and in-hospital mortality models showed reduced net benefit in the external cohort, indicating that statistical performance does not necessarily translate into clinical utility. The small size of the prospective cohort further limits the stability of these estimates. Larger multicenter validations, recalibration analyses, and assessment of decision-analytic performance are therefore required before routine clinical implementation.

## 4. Materials and Methods

### 4.1. Patient Cohort

We analyzed data from randomly selected patients with suspected sepsis admitted to Fondazione Policlinico Universitario Campus Bio-Medico between 2020 and 2024. The study followed the Declaration of Helsinki and received approval from the Ethical Committee of the University Hospital Campus Bio-Medico of Rome (28.16TS Com Et CBM). Approval date 23 June 2016. All patients provided informed consent at hospital admission.

We diagnosed sepsis and septic shock using the criteria of the Third Consensus Conference. Patients met these criteria when infection was present and their q-SOFA or SOFA score increased by at least two points from baseline. Clinical management followed the recommendations of the Third Consensus and its subsequent update [1,14].

For each patient, we collected the following information:

- Demographic characteristics: age, gender, and smoking status, used to describe the patient’s general health profile.
- Clinical data: comorbidities, nutritional status, in-hospital mortality, and mortality at 30 and 90 days, describing the patient’s clinical condition and outcomes.
- Clinical scores: q-SOFA, SOFA, Glasgow Coma Scale (GCS), and SIRS, used to evaluate illness severity.
- Laboratory biomarkers: sepsis-related markers such as CRP, PCT, MR-proADM, and lactate, reflecting metabolic state, inflammation, and sepsis burden.

The prospective control group (PRO) consisted of hospitalized patients without sepsis or septic shock according to Sepsis-3 criteria, admitted for other clinical conditions. This design reflects a real-world diagnostic setting, in which the clinical challenge lies in distinguishing septic from non-septic acutely ill patients rather than from healthy individuals.

### 4.2. Machine Learning Training Pipeline

**Data Preprocessing:** All datasets followed a unified preprocessing pipeline to ensure data quality and consistency before model training. Numerical features were standardized using z-score normalization, and categorical variables were transformed through one-hot encoding. To prevent data leakage, all preprocessing steps were fitted exclusively on the training set. Specifically, the parameters required for normalization (mean and standard deviation) and the encoding scheme for categorical variables were learned from the training data only. The same fitted transformations were subsequently applied to the validation and test cohorts without refitting. This procedure ensured that no information from the test data influenced model training or preprocessing.

For the validation approach, we use an 80:20 hold-out split, assigning 80% of the data to training and 20% to testing. For all tasks, stratified sampling preserved class proportions in both sets, an essential step when working with imbalanced datasets. From the training portion, a further 10% split was extracted to create a validation subset.

**Machine Learning Training:** A systematic training process was implemented involving 25 distinct machine learning classification algorithms, encompassing a diverse range of model types such as ensemble methods, linear models, kernel-based methods, probabilistic classifiers, and neural networks. The training was conducted using the training set, where each model was trained using default hyperparameters provided by the scikit-learn and XGBoost libraries. Model selection was conducted on the validation subset using the MCC as the primary metric. MCC was chosen due to its robustness in imbalanced classification settings, as it integrates all four entries of the confusion matrix (true positives, true negatives, false positives, and false negatives), providing a balanced assessment beyond accuracy or F1-score.

**Algorithms Considered:** The following machine learning algorithms were selected and categorized based on their methodological approach:

- Ensemble Methods: AdaBoost Classifier, ExtraTrees Classifier, Gradient Boosting Classifier, Histogram Gradient Boosting Classifier, Random Forest Classifier, XGBoost Classifier, XGBoost Random Forest Classifier.
- Naive Bayes Classifiers: Bernoulli Naive Bayes, Gaussian Naive Bayes
- Tree-Based Methods: Decision Tree Classifier, ExtraTrees Classifier.
- Linear Models: Logistic Regression, Ridge Classifier, Passive Aggressive Classifier, Perceptron, SGD Classifier.
- Support Vector Machines: Support Vector Machine, Linear Support Vector Machine.
- Nearest Neighbors: K-Nearest Neighbors Classifier.
- Neural Networks: Multi-Layer Perceptron Classifier.
- Discriminant Analysis: Linear Discriminant Analysis, Quadratic Discriminant Analysis.
- Gaussian Processes: Gaussian Process Classifier.
- Semi-Supervised Methods: Label Propagation, Label Spreading.

Each algorithm was implemented using the scikit-learn python library, ensuring reproducibility and consistency across the models.

**Evaluation of the Best Model:** We conducted a detailed assessment of the top-performing model using complementary evaluation metrics to capture its predictive behavior:

Area Under the ROC Curve (AUC-ROC) that quantifies the model’s ability to discriminate between classes across all decision thresholds; Matthews Correlation Coefficient (MCC): offers a balanced indicator of performance, especially in the presence of class imbalance, by integrating all entries of the confusion matrix.

ROC curves were generated to examine performance across threshold settings and to visualize the trade-off between sensitivity and specificity. To contextualize the model’s performance, we compared the best-performing classifier with a baseline Logistic Regression model, highlighting the gain in AUC. We also tested the statistical significance of this improvement using the DeLong test, which evaluates differences between correlated ROC curves.

**Explainable Artificial Intelligence (XAI):** To improve interpretability and clarify the model’s underlying decision process, we applied SHAP (SHapley Additive exPlanations). SHAP values attribute each prediction to the contribution of individual features, offering a unified and model-agnostic view of feature importance. We generated SHAP summary plots to visualize the distribution and direction of feature effects. This analysis highlighted the variables that most strongly influenced the model’s predictions and helped verify that the model’s behavior aligned with clinical expectations. Such transparency supports bias detection, regulatory compliance, and domain-specific interpretation, while also offering insights that may guide future clinical investigations.

### 4.3. Decision Curve Analysis

To evaluate the clinical applicability and transportability of the selected machine learning models, an external validation was performed on a prospective cohort of newly enrolled patients. This cohort was completely independent from the development dataset used for model training and the testing (indicated as DEV).

Beyond conventional discrimination metrics, clinical utility was assessed using Decision Curve Analysis (DCA). DCA estimates the Net Benefit (NB) of a predictive model across a range of decision threshold probabilities and compares it with default management strategies, namely treating all patients or treating none. Net Benefit was calculated as

$$NB=\frac{TP}{N}-\frac{FP}{N}\cdot \frac{p_{t}}{1-p_{t}}$$

where TP denotes true positives, FP false positives, N the total number of patients, and $p_{t}$ the threshold probability. This formulation incorporates the relative clinical consequences of false-positive and false-negative decisions.

For each prediction task defined within the PRO cohort, sepsis, septic shock, and in-hospital mortality, net benefit curves are generated in both the DEV cohort and the PRO cohort. The optimal operating threshold was defined as the threshold probability associated with the highest net benefit. The incremental Net Benefit (iNB) was computed as the difference between the model’s net benefit and the best-performing default strategy at the same threshold. This framework allowed assessment of the model’s potential to improve decision-making beyond statistical discrimination alone.

## 5. Conclusions

In conclusion, our study highlights the potential of ML-based approaches to enhance risk stratification in sepsis across its clinical continuum, from early detection to long-term survival prediction. By leveraging biomarkers, clinical scores, and patient characteristics, ML algorithms can achieve superior performance compared to traditional statistical models, while maintaining clinical interpretability through SHAP analysis. Despite these limitations, the modeling task provides proof-of-concept evidence that predictive algorithms can assist in identifying patients at risk of deterioration, supporting timely intervention and more personalized management strategies. These results pave the way for future prospective validation and integration of ML-based decision support systems into routine sepsis care, ultimately improving patient outcomes and optimizing resource allocation.

## Figures and Tables

**Table 1:** Characteristics of study population: demographic characteristics, clinical scores and biomarkers of the study population classified as patients with sepsis and septic shock and control.

Variables	Control N = 126		Patients with Sepsis N = 251		Patients with Septic Shock N = 100	
	Mean (SD)	Median (IQR)	Mean (SD)	Median (IQR)	Mean (SD)	Median (IQR)
Age in years, median	74 (19)	80 (68–87)	71 (13)	73 (65–80)	73 (12)	76 (68–82)
Sex category, number female (%)	62 (49%)	-	119 (47%)	-	49 (49%)	-
SOFA	1.86 (1.32)	2.00 (1.00–3.00)	4.39 (2.98)	4.00 (2.00–6.00)	6.07 (3.17)	6.00 (4.00–8.00)
q-SOFA	0.16 (0.39)	0(0.00–0.00)	1.44 (0.99)	1.00 (1.00–2.00)	1.95 (0.83)	2.00 (1.00–3.00)
GCS	-	-	12.85 (3.06)	14.00 (12.00–15.00)	11.90 (3.51)	13.00 (10.00–15.00)
MR-proADM, nmol/L	1.51 (0.98)	1.19 (0.84–1.86)	3.87 (3.37)	2.79 (1.89–4.51)	5 (4.34)	3.69 (2.13–6.35)
PCT, ng/mL	0.42 (0.98)	0.10 (0.05–0.30)	11.55 (35.14)	1.25 (0.43–5.58)	17.97 (47.75)	1.60 (0.73–9.10)
CRP mg/L	14.99 (24.46)	8.43 (2.33–16.40)	123.41 (96.66)	109.82 (49.13–175.98)	115.78 (94.12)	98.76 (52.78–157.51)
Lactate mmol/L	-	-	16.44 (12.88)	13.00 (9.58–19.43)	23.26 (16.65)	19.00 (12.90–28.50)

## Data Availability

Data is unavailable due to privacy and ethical restrictions. The analysis code for the methodology developed in this study is available upon request from the corresponding author.
